# Assessment of retroperitoneal lymph node status in locally advanced cervical cancer

**DOI:** 10.1186/s12885-021-08208-6

**Published:** 2021-05-01

**Authors:** Wei Li, Li Xiong, Qiaoling Zhu, Hong Lu, Meiling Zhong, Meirong Liang, Wei Jiang, Yanan Wang, Wei Cheng

**Affiliations:** 1 Department of Gynecology, Hunan Maternal and Child Health Hospital, Changsha, Hunan China; 2 Department of Pathology, Hunan Maternal and Child Health Hospital, Changsha, Hunan China; 3 Department of Oncology, Jiangxi Maternal and Child Health Hospital, Nanchang, Jiangxi China

**Keywords:** Retroperitoneal lymph node, Cervical neoplasm, Computed tomography (CT), Squamous cell carcinoma antigen (SCC-Ag)

## Abstract

**Background:**

The assessment of retroperitoneal lymph node status in patients with locally advanced cervical cancer is still a problem. This study aimed to explore the choice of these assessment methods.

**Methods:**

Laparoscopic retroperitoneal lymphadenectomy was performed in 96 patients with advanced cervical cancer. The positive rates of lymph node metastasis were analyzed. The values of computed tomography lymph node minimum axial diameter (MAD) and squamous cell carcinoma antigen (SCC-Ag), and their combination in predicting retroperitoneal lymph node metastasis were compared. High-risk factors for common iliac lymph node (CILN) and/or para-aortic lymph node (PALN) metastasis were analyzed.

**Results:**

The lymph node metastasis rate was 62.50% and the CILN and/or PALN metastasis rate was 31.25%. Overall, 96 patients had 172 visible lymph nodes. The positive rate of lymph node metastasis was significantly higher in the MAD ≥1.0 cm group (83.33%) than in the 0.5 cm ≤ MAD < 1.0 cm group (26.82%). The critical values of MAD and SCC-Ag in determining lymph node metastasis were 1.0 cm and 5.2 ng/mL, respectively. The accuracy, specificity, and Youden index of MAD ≥1.0 cm combined with SCC-Ag ≥ 5.2 ng/mL for evaluating lymph node metastasis were 75.71%, 100%, and 0.59, respectively, and were significantly different from the values for the MAD ≥1.0 cm (72.09%, 80.56%, and 0.47, respectively) and SCC-Ag ≥ 5.2 ng/mL (71.43%, 68.97%, and 0.42, respectively) groups. Correlation analysis showed that non-squamous cell carcinoma, pelvic lymph node (PLN) MAD ≥1.0 cm plus number ≥ 2, and 1 PLN MAD ≥1.0 cm with CILN and/or PALN MAD 0.5–1.0 cm were risk factors for CILN and/or PALN metastasis.

**Conclusion:**

Patients with MAD ≥1.0 cm and SCC-Ag ≥ 5.2 ng/mL, as well as high risk factors for CILN and/or PALN metastasis, should undergo resection of enlarged lymph nodes below the common iliac gland and lymphadenectomy of CILN/PALN to reduce tumor burden and to clarify lymph node metastasis status for accurate guidance in follow-up treatment. Patients with MAD < 1.0 cm and SCC-Ag < 5.2 ng/mL may be treated with chemoradiotherapy directly based on imaging, given the low lymph node metastasis rate.

## Background

In 2018, the International Federation of Gynecology and Obstetrics (FIGO) officially included the status of lymph node metastasis in the clinical staging of cervical cancer [1]. Lymph node metastasis is one of the independent prognostic factors for cervical cancer and the lymph node metastasis rate directly parallels the clinical stage. For example, the probabilities of pelvic lymph node (PLN) and para-aortic lymph node (PALN) metastases in patients with stage IB are 11–20% and 2–7%, respectively, and these increase in phase II to 36–45% and 7.2–25%, respectively, while phase III levels reach as high as 40–71% and 21–37%, respectively [2–4]. Patients with common iliac lymph node (CILN) and/or PALN metastasis are treated by extended abdominal radiotherapy in the para-aortic lymphatic drainage area [5]. Concurrent chemoradiotherapy is the standard treatment mode for locally advanced cervical cancer. The radiotherapy dose and radiation field range determine the therapeutic effect, but inadequate sensitivity and specificity can limit the use of radiotherapy and chemotherapy.

The lymph node status is typically evaluated by computed tomography (CT), magnetic resonance imaging (MRI), and positron emission tomography/computed tomography (PET-CT) [6]. The sensitivity of CT for detecting metastatic lymph nodes is only about 60%. By contrast, the sensitivity and specificity of MRI for judging parametrial infiltration and bladder and rectum invasion are good, but the diagnostic value of metastatic lymph node imaging is limited, with a sensitivity of only 30% [7–9]. Similarly, even the most accurate diagnosis of lymph nodes by PET-CT still has a high false-positive rate [10]. Therefore, the broad use of imaging-guided radiotherapy inevitably has problems [11].

When conducted prior to radiotherapy treatment, laparoscopic retroperitoneal lymphadenectomy can clarify the status of lymph node metastasis, remove the enlarged lymph nodes that are difficult to cure by radiotherapy, provide accurate guidance for the follow-up treatment of patients, and allow a more accurate assessment of the patient’s prognosis [12, 13]. However, retroperitoneal lymphadenectomy increases the patient’s surgical complications and hospitalization costs to some extent [14, 15]. Therefore, the decision to perform retroperitoneal lymph node resection before radiotherapy needs to be individualized with an auxiliary examination. How to shunt patients with locally advanced cervical cancer according to the auxiliary examination is an urgent problem to be solved. The aim of the present study was to investigate the available options for assessing the status of retroperitoneal lymph node status in locally advanced cervical cancer to ensure optimal treatment.

## Methods

### Clinical data

This study was approved by the Ethics Committee of the Hunan Maternal and Child Health Hospital. All patients provided signed informed consent.

**Fig. 1:**
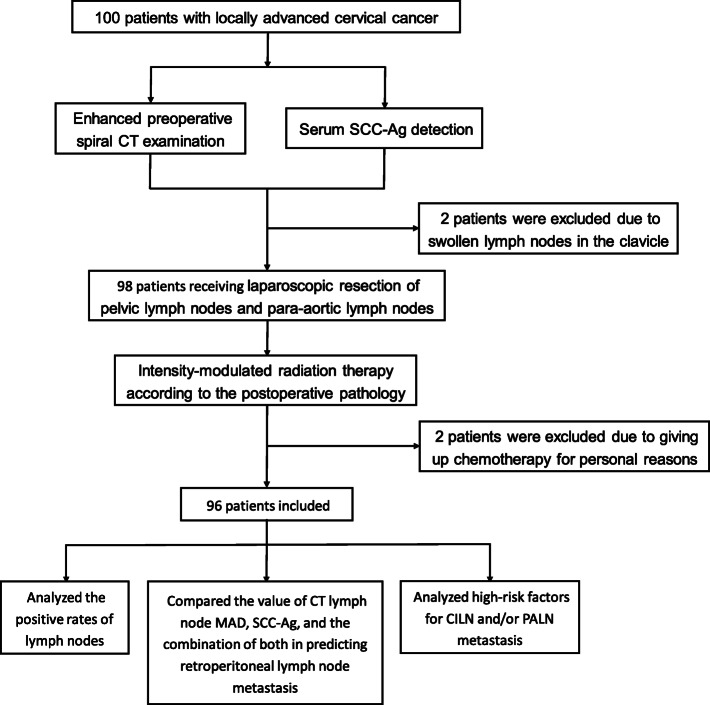
Flowchart of the study. CT, computed tomography; SCC-Ag, squamous cell carcinoma antigen; MAD minimum axial diameter; CILN, common iliac lymph node; PALN, para-aortic lymph node

A total of 96 patients with locally advanced cervical cancer who underwent laparoscopic retroperitoneal lymphadenectomy at the Oncology Department of Hunan Maternal and Child Health Hospital from March 2011 to October 2018 were enrolled. The patient age ranged from 23 to 65 years, with a median age of 48 years. The cases included 12 IB2 stage, three IIA2 stage, 48 IIB stage, two IIIA stage, and 31 IIIB stage cervical cancers. In addition, the study population included 86 cases of squamous cell carcinoma and 10 cases of non-squamous cell carcinoma (five cases of adenocarcinoma, four cases of adenosquamous cell carcinoma, and one case of neuroendocrine small cell carcinoma). A flowchart of the study is shown in Fig. 1.

### Inclusion criteria

The patients were included in the study if they had stage IB2–IIIB cervical cancer (FIGO 2009); had not undergone any preoperative radiotherapy, chemotherapy or operative contraindication; had signed an informed consent form; had undergone a comprehensive preoperative assessment (i.e., medical history inquiry, physical examination, squamous cell carcinoma-related antigen [SCC-Ag] detection, and imaging examination); and had their preoperative and postoperative pathology confirmed by two senior pathologists.

### Exclusion criteria

Patients were excluded from the study if they had undergone preoperative radiotherapy or chemotherapy or had distant metastasis indicated by imaging.

### Surgical methods

We performed laparoscopic resection of the PLNs and PALNs [16, 17]. The upper boundary of PLN resection was the abdominal aorta bifurcation, and the lower boundary was the deep circumflex iliac vein across the external iliac artery. PALN resection ranged from the abdominal aorta bifurcation to the area of the inferior mesenteric artery. The lymph nodes were marked according to the surgical site and then sent for examination. Each lymph nodes were sectioned and examined by routine hematoxylin and eosin (H&E) staining for presence/absence of lymph node metastasis.

### Enhanced preoperative spiral computed tomography examination

Before the operation, all patients underwent whole abdominal enhanced spiral CT with a Siemens 16-slice spiral CT system. The scanning thickness and interval were 5 mm, and the scanning range extended from the diaphragm top to the vulva area. A reinforcing agent consisting of 70–90 mL of non-ionic contrast agent was injected via the elbow vein at a rate of 3.5 mL/s, with delayed scanning of 30 s [18].

### CT evaluation method

The CT imaging data of all patients (96 cases) were reviewed by radiologists and radiotherapists. The PLNs and PALNs were grouped based on the Radiation Therapy Oncology Group (RTOG) classification. The PLNs were divided into left and right sides, and each side was separated into five parts: total iliac, internal iliac, external iliac, presacral, and obturator. The minimum axial diameter (MAD) of the PALN and PLN cross-section was measured. The retroperitoneal lymph nodes detected on CT images were compared with the postoperative pathology. The pathological findings of retroperitoneal lymph nodes were used as the gold standard to judge the accuracy of CT diagnosis [15, 16].

### Serum SCC-Ag detection

A total of 3 mL of venous blood was collected before treatment. Serum SCC-Ag concentration was detected by microparticle enzyme immunoassay using Abbott’s fully automatic fluorescent enzyme label analyzer. Briefly, in the first step, the sample and the SCC antibody-coated paramagnetic microparticles were mixed and the SCC antigen in the sample was allowed to bind to the SCC antibody-coated microparticles. After rinsing, an acridinium ester–labeled SCC antibody conjugate was added to form a reaction mixture. After further rinsing, the pre-excitation liquid and the excitation liquid were added to the reaction mixture. The resulting chemiluminescent reactions were measured and expressed as relative luminescence units (RLUs). The SCC antigen in the sample was directly proportional to the RLU value detected by the optical system [19].

### Radiotherapy

All patients underwent Intensity-Modulated Radiation Therapy (IMRT) according to their postoperative pathology. The target area for radiotherapy was delineated according to the consensus of the target area for RTOG cervical cancer. The IMRT plan was designed using the Pinnacle planning system (Philips, Netherlands, version 9.7).

### Statistical analysis

All data were analyzed using the Chi-squared (χ2) test in the SPSS (Version 17.0) statistical software. *P* < 0.05 was considered statistically significant.

## Results

### Intraoperative and postoperative indicators of surgery

The duration of surgery was 1.5–3 h, with an average of 2 h; the bleeding volume was 10–100 mL, with an average of 40 mL; radiotherapy was started 6 to 13 d postoperatively, with an average of 9.2 d; postoperative complications included two cases of pure cyst, one case of lymphocyst with lower extremity edema, and six cases of lymphocyst associated with infection.

### Lymph node metastasis

A total of 2937 PLNs were removed from the 96 patients, with an average number of 30.6 per patient, (range 17–53). Similarly, 758 PALNs were removed, with an average number of 7.9 per patient (range 6–22). The lymph node metastasis rate was 62.5% (60/96) and the CILN and/or PALN metastasis rate was 31.2% (30/96). Only 15 cases showed a MAD for CILN and/or PALN of more than 0.5 cm. In 86 cases of squamous cell carcinoma, the lymph node metastasis rate was 58.1% (50/86) and the CILN and/or PALN metastasis rate was 24.4% (21/86); the latter rate included one case of PALN metastasis alone, two cases of CILN metastasis alone, and 18 cases of PLN metastasis below the total iliac. In 10 cases of non-squamous cell carcinoma, the tumor markers (CA125, CEA, and SCC-Ag) were within the normal range (data not shown); CT showed five cases of MAD ≥1 cm in the retroperitoneal lymph node, 0.5 cm ≤ MAD < 1.0 cm in three cases, and MAD < 0.5 cm in two cases, but the lymph nodes were extensively metastasized after the operation, and nine cases showed CILN and/or PALN metastasis. One case had metastasis of the mesorectal surface and right oviduct.

### Positive rate of retroperitoneal lymph node metastasis judged by CT images of different MAD of lymph nodes

**Fig. 2:**
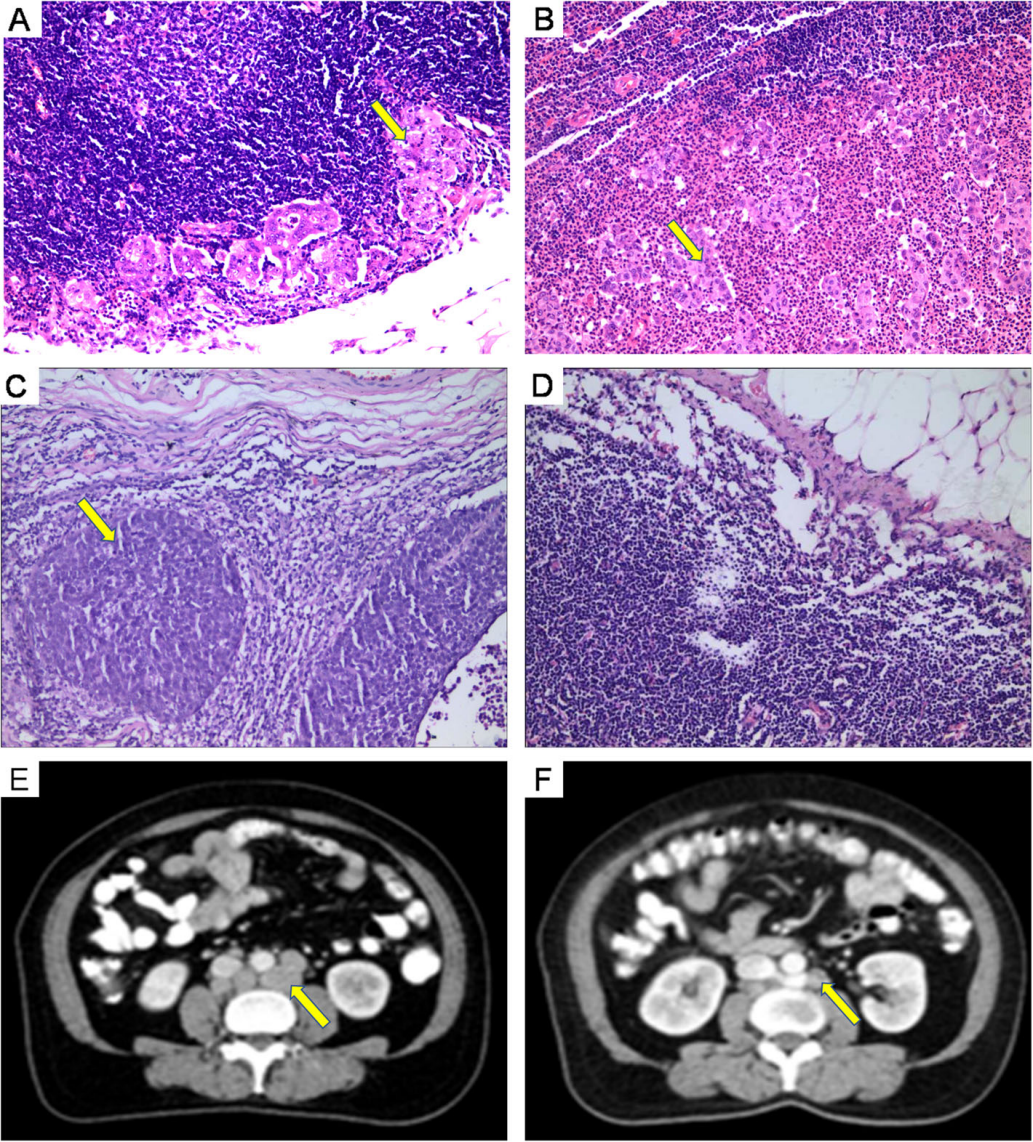
Computed tomography (CT) scans and postoperative hematoxylin and eosin (H&E) staining of responding lymph node. **a**. Lymph node metastasis of adenocarcinoma (yellow arrow) comfirmed by H&E staining. **b**. Lymph node metastasis of adenosquamous carcinoma (yellow arrow) comfirmed by H&E staining. **c**. Lymph node metastasis of squamous cell carcinoma (yellow arrow) comfirmed by H&E staining. **d**. Normal lymph nodes. **e**. Measurement of MAD (≥ 1.0 cm) of the para-aortic lymph node. The yellow arrows showed the location of lymph node metastasis. The H&E staining result was positive. **f**. Measurement of MAD (0.5–0.9 cm) of the para-aortic lymph node. The yellow arrows showed the location of lymph node metastasis. The H&E staining result was negative

CT revealed 172 visible lymph nodes in 96 patients. The postoperative pathology was used as the gold standard for judging retroperitoneal lymph node metastasis (Fig. 2). The MAD of the CT lymph nodes was used to determine the retroperitoneal lymph node metastasis (Fig. 2). The MAD was used to divide patients into a 0.5 cm ≤ MAD < 1.0 cm group and a MAD≥1.0 cm group. The positive rate of lymph node metastasis was higher in the MAD ≥1.0 cm group (83.33%) than in the 0.5 cm ≤ MAD < 1.0 cm group (26.82%), and the difference was statistically significant (*P* < 0.05) (=Table 1).

**Table 1:** Comparison of positive rates of retroperitoneal lymph node metastasis judged by differences in computed tomography (CT) lymph node minimum axial diameter (MAD)

Group	Number	Pathological results		Percentage (%)	*P* value
		Positive	Negative		
MAD ≥1.0 cm	78	65	13	83.33	0.000
0.5 cm ≤ MAD < 1.0 cm	82	22	60	26.82	

### Critical value of CT lymph node MAD and serum SCC-Ag levels for evaluating retroperitoneal lymph node metastasis of cervical squamous cell carcinoma

**Fig. 3:**
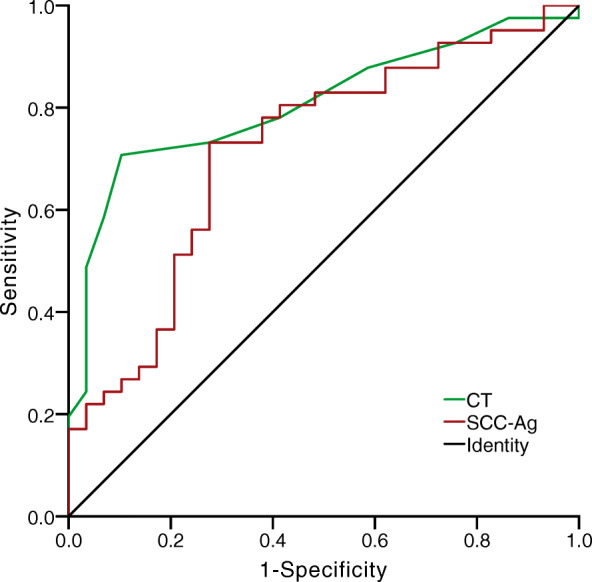
The critical values of computed tomography (CT) lymph node minimum axial diameter (MAD) (AUC = 0.782) and serum squamous cell carcinoma antigen (SCC-Ag) (AUC = 0.717) levels in evaluating retroperitoneal lymph node metastasis of cervical squamous cell carcinoma

Of the 86 cases with cervical squamous cell carcinoma, 16 cases did not undergo SCC-Ag detection before the operation. The area under the receiver operating characteristic (ROC) curve for the CT lymph node MAD was 0.782 (95% CI 0.706–0.859). The Youden index was the largest when the CT lymph node MAD was 1.0 cm. The area under the ROC curve for serum SCC-Ag levels was 0.717 (95% CI 0.594–0.841). The Youden index was the largest (0.456) when SCC-Ag was 5.2 ng/mL (Fig. 3). Spearmen correlation analysis of MAD ≥1.0 cm and SCC-Ag ≥ 5.2 ng/mL was performed. The result suggested that the combination of these two indicators may further improve the predictive validity of lymph node metastasis.

### Efficacy of preoperative CT and/or serum SCC-Ag in assessing retroperitoneal lymph node metastasis of cervical squamous cell carcinoma

The accuracy, specificity, and Youden index for a CT lymph node MAD ≥1.0 cm, together with SCC-Ag ≥ 5.2 ng/mL, were 75.71%, 100%, and 0.59, respectively, and these values were significantly different from those of the CT lymph node MAD ≥1.0 cm group (72.09%, 80.56%, and 0.47, respectively) and the SCC-Ag ≥ 5.2 ng/mL group (71.43%, 68.97%, and 0.42, respectively) (Table 2**,** all *P <* 0.05). Twenty-four patients with CT lymph node MAD ≥1.0 cm and SCC-Ag ≥ 5.2 ng/mL (the double positive group) had lymph node metastasis after the operation, and 50% (12/24) showed CILN and/or PALN metastasis. Eight patients with CT lymph node MAD ≥1.0 cm and SCC-Ag < 5.2 ng/mL (the CT positive group) had CT positive lymph node metastasis, and five of the eight had postoperative lymph node metastasis. Fifteen patients with CT lymph node MAD < 1.0 cm and SCC-Ag ≥ 5.2 ng/mL (SCC-Ag positive group) had SCC-Ag positive lymph node metastasis, and six of these showed postoperative lymph node metastasis. Among the twenty-three cases with CT lymph node MAD < 1.0 cm and SCC-Ag < 5.2 ng/ml, only six cases (26.08 %) had PLN metastasis below CILN after the operation. The double positive group was significantly different from the other groups (all *P* < 0.05).

**Table 2:** Efficacy of CT lymph node MAD and/or serum SCC-Ag in judging retroperitoneal lymph node metastasis in patients with locally advanced cervical squamous cell carcinom

Group	Case	Pathological results		Accuracy (%)	Sensitivity(%)	Specificity (%)	Youden index
		Positive	Negative				
CT lymph node MAD ≥1.0 cm + SCC-Ag ≥ 5.2 ng/mL	24	24	0	75.71*	58.54	100.0*	0.59*
CT lymph node MAD ≥1.0 cm	40	33	7	72.09	66.0	80.56	0.47
SCC-Ag ≥ 5.2 ng/mL	39	30	9	71.43	73.17	68.97	0.42

*CT* computed tomography, *MAD* minimum axial diameter, *SCC-Ag* Squamous cell carcinoma antigen. *P  < 0.05 vs. the CT lymph node MAD ≥1.0 cm group and SCC-Ag ≥ 5.2 ng/mL group

### High-risk factors for CILN and/or PALN metastasis in locally advanced cervical cancer

Among the 96 patients, CT showed CILN and/or PALN MAD 0.5 to 1.0 cm in 11 patients, including seven patients with postoperative CILN and/or PALN metastases. CT showed CILN and/or PALN MAD ≥1.0 cm in 8 cases, and all patients showed postoperative CILN and/or PALN metastases. Correlation analysis showed that non-squamous cell carcinoma, PLN MAD ≥1.0 cm plus number ≥ 2, and 1 PLN MAD ≥1.0 cm with CILN and/or PALN MAD 0.5–1.0 cm were risk factors for CILN and/or PALN metastasis (all *P* < 0.05) (Table 3).

**Table 3:** The relationship between clinical pathological features of advanced cervical cancer and CILN and/or PALN metastasis

Factors	Case	Post-operation CILN and/or PALN		χ^2^value	*P* value
		Positive	Negative		
Clinical stages					
IB2 stage	12	5	7		
IIA2 - IIB stage	51	15	36	0.700	0.705
IIIA - IIIB stage	33	10	23		
Pathological type					
Squamous cell carcinoma	86	21	65	15.011	0.000
Non-Squamous cell carcinoma	10	9	1		
Tumor diameter (cm)					
≤ 4	33	11	22	0.102	0.750
> 4	63	19	44		
SCC-Ag (ng/mL)					
≥ 5.2	39	14	25	3.653	0.056
< 5.2	31	4	27		
CT indicates PLN MAD (cm)^a^					
< 0.5	7	1	6	1.315	0.518
0.5–0.9	34	5	29		
≥ 1.0	36	9	27		
CT indicates PLN MAD 0.5–1.0 cm (number) ^a^					
1	24	4	20	–	1.000
≥ 2	10	1	9		
CT indicates PLN MAD ≥1.0 cm (number)^a^					
1	26	3	23	9.046	0.003
≥ 2	10	6	4		
CT indicates one PLN MAD ≥1.0 cm ± MAD 0.5–1.0 cm CILN and/or PALN					
Yes	3	3	0	_	0.005
No	26	3	23		

*CILN* common iliac lymph node, *PALN* para-aortic lymph node, *PLN* pelvic lymph node, *SCC-Ag* squamous cell carcinoma antigen, *CT* computed tomography, *MAD* minimum axial diameter. *P* < 0.05 was considered statistically significant. ^a^ Did not include patients whose CT showed CILN and/or PALN MAD≥0.5 cm

## Discussion

Lymph node metastasis is an independent prognostic factor for cervical cancer. Although CT, MRI, and PET-CT have been widely used to evaluate the status of lymph nodes, imaging has several disadvantages, including insufficient sensitivity and accuracy; therefore, imaging-based radiotherapy has a certain degree of under- or over-treatment [6–11]. Surgical staging before radiotherapy can confirm lymph node metastasis and provide accurate guidance for follow-up treatment, but it increases the operative complications and hospitalization costs; therefore, the impact on prognosis needs further research [14, 15]. For these reasons, the assessment of retroperitoneal lymph node status of locally advanced cervical cancer needs to be individualized, and strategies need to be developed for shunting patients with locally advanced cervical cancer determined by auxiliary examinations.

Serum SCC-Ag is an antigen with high specificity for the diagnosis of squamous cell carcinoma. Clinical diagnosis and prognosis of patients with squamous cell carcinoma are usually determined by the levels of SCC-Ag. For example, Duk studied 653 patients with squamous cervical carcinoma and found that the level of SCC-Ag was positively related to lymph nodes. The sensitivity and specificity of SCC-Ag > 1.9 ng/ml was 64.6% and 68.4%, respectively [20]. Another study suggested that SCC-Ag > 4 ng/ml would raise the probability of lymph node metastasis by 4.2 folds (OR = 4.212, *P* < 0.05) [21]. Zhou et al. performed a meta-analysis of the value of serum SCC-Ag levels in the diagnosis of the lymph nodal metastasis in cervical cancer [22]. They found that the area under the ROC curve of SCC-Ag for pelvic lymph node metastasis was 0.71, and the best cutoff value was 2.15 ng/mL, with a sensitivity of 69.7% and a specificity of 66.4% [22]. In the present study, the area under the ROC curve of serum SCC-Ag levels in the retroperitoneal lymph node metastasis was 0.717. When the SCC-Ag was 5.2 ng/ml, the Youden index was the largest (0.456), and the corresponding sensitivity and specificity were 73.17. % and 68.97%, respectively. Taken together, the findings of these studies indicate that SCC-Ag has a certain diagnostic value for lymph node metastasis of cervical squamous cell carcinoma. However, it is not ideal and needs to be combined with other indicators.

The combination of preoperative CT and serum SCC-Ag levels has been found to have a greater value in assessing retroperitoneal lymph node metastasis in a previous study of 197 cervical squamous cell carcinoma patients by Xu [23]. This study also showed that the combination of CT and SCC-Ag levels was a better way to evaluate the status of the retroperitoneal lymph node. In the present study, the accuracy, specificity, and Youden index of CT lymph node MAD (≥ 1.0 cm) with SCC-Ag (≥ 5.2 ng/mL) were 75.71%, 100%, and 0.59, respectively. Twenty-four patients in the group with CT lymph node MAD ≥1.0 cm combined with SCC-Ag ≥ 5.2 ng/mL had metastasis rate of 100%, whereas only six of 23 patients in the group with CT lymph node MAD < 1.0 cm and SCC-Ag < 5.2 ng/mL had PLN metastasis below CILN. Therefore, patients with CT lymph node MAD ≥1.0 cm and SCC-Ag ≥ 5.2 ng/mL should be treated with lymphadenectomy to clarify their lymph node metastasis status and provide accurate guidance for subsequent treatment. Patients with CT lymph node MAD < 1.0 cm and SCC-Ag < 5.2 ng/mL had a low lymph node metastasis rate, so they can be treated with radiotherapy and chemotherapy directly based on imaging.

For the extent of resection of retroperitoneal lymph nodes for locally advanced cervical cancer, as retroperitoneal lymph node surgery is pelvic and para-aortic lymph node dissection with a large surgical field, which can lead to a longer operative time and an increased probability of complications, so lymph nodes below the total iliac with MAD < 1.0 cm are not considered for routine inclusion in the resection range as they are located in the conventional treatment field [12, 13]. However, for enlarged lymph nodes with MAD > 1.0 cm, the radical dose should be 60–65 Gy, whereas for lymph nodes with MAD > 2.0 cm, the radical dose should be 65–70 Gy. Lymph nodes with MAD > 3.0 cm are difficult to cure by radiotherapy alone. Cosin et al. retrospectively analyzed 266 patients with cervical cancer who underwent retroperitoneal lymph node resection before radiotherapy [24]. They found comparable 10-year and 5-year disease-free survival rates for patients with enlarged or with small metastatic lymph node resection, and those patients who did not have enlarged lymph nodes removed all had recurrences. Other studies have shown that the removal of swollen positive lymph nodes is beneficial to the survival of patients with cervical cancer [25]. Therefore, we recommend the resection and sampling of enlarged lymph nodes below the common iliac gland with the MAD greater than 1.0 cm.

For CILN and/or PALN, the metastatic status will determine the radiotherapy regimen. A comprehensive assessment should be performed based on preoperative auxiliary examinations, such as imaging data and tumor markers. Shim et al. showed that the diagnosis of PALN metastasis by PET-CT and tumor diameter ≥ 5 cm were independent factors affecting PALN metastasis [26]. His recommendation is that patients with a tumor diameter of ≥5 cm and PET-CT indicative of PALN metastasis should undergo the PALN resection, while radiotherapy and chemotherapy can be used directly in the low-risk and high-risk groups. In the present study, non-squamous cell carcinoma, PLN MAD ≥1.0 cm plus number ≥ 2, and 1 PLN MAD ≥1.0 cm with CILN and/or PALN MAD 0.5–1.0 cm were risk factors for CILN and/or PALN metastasis, and our recommendation was resection of the lymph nodes below the common iliac gland and CILN/PALN. For PLNs with a MAD of 0.5–1.0 cm below the common iliac gland, the CILN and/or PALN metastasis rate did not change significantly regardless of the number of lymph nodes or the existence of CILNs and/or PALNs with MAD between 0.5–1.0 cm, so these PLNs can be directly treated with chemoradiotherapy according to the imaging. PALN also did not require extended field radiotherapy to avoid adverse reactions.

The present study has some limitations. One is that the sample size was small, and the operation was performed with PLN and PALN resection. Further study is also needed in patients with only one CT lymph node MAD ≥1.0 cm and the serum SCC-Ag < 5.2 ng/mL, as well as SCC-Ag ≥ 5.2 ng/mL and retroperitoneal lymph node MAD < 1.0 cm, to determine whether PET-CT should be performed first to understand lymph node metastasis to determine the subsequent treatment. The effect of surgical staging on subsequent chemotherapy and prognosis also needs further study to confirm the safety and feasibility of laparoscopic retroperitoneal lymph node dissection, so that patients will experience an effective treatment and have a longer survival time while enjoying a good quality of life.

## Conclusions

The patients with CT lymph node MAD ≥1.0 cm and SCC-Ag ≥ 5.2 ng/mL, as well as high risk factors for CILN and/or PALN metastasis, should undergo resection of enlarged lymph nodes below the common iliac gland, as well as lymphadenectomy of CILN/PALN to reduce tumor burden and to clarify lymph node metastasis status to provide accurate guidance for the follow-up treatment. Patients with CT lymph node MAD < 1.0 cm and SCC-Ag < 5.2 ng/mL may be treated with chemoradiotherapy directly based on imaging, given the low lymph node metastasis rate in these patients.

## Data Availability

The data that support the findings of this study are available from the corresponding author upon reasonable request.

## Abbreviations

FIGO: International Federation of Gynecology and Obstetrics
PLN: Pelvic lymph node
PALN: Para-aortic lymph node
CILN: Common iliac lymph node
CT: Computed tomography
MRI: Magnetic resonance imaging
PET-CT: Positron emission tomography/computed tomography
SCC-Ag: Squamous cell carcinoma antigen
H&E: Hematoxylin and eosin
RTOG: Radiation Therapy Oncology Group
MAD: Minimum axial diameter
RLUs: Relative luminescence units
IMRT: Intensity-Modulated Radiation Therapy
ROC: Receiver operating characteristic
